# ES-YOLO: Multi-Scale Port Ship Detection Combined with Attention Mechanism in Complex Scenes

**DOI:** 10.3390/s25247630

**Published:** 2025-12-16

**Authors:** Lixiang Cao, Jia Xi, Zixuan Xie, Teng Feng, Xiaomin Tian

**Affiliations:** 1 School of Remote Sensing and Information Engineering, North China Institute of Aerospace Engineering, Langfang 065000, China; caolixiang1@stumail.nciae.edu.cn (L.C.); xijia069@163.com (J.X.); xiezixuan1113@163.com (Z.X.); teng_f2024@163.com (T.F.); 2 Hebei Collaborative Innovation Center for Aerospace Remote Sensing Information Processing and Application, Langfang 065000, China

**Keywords:** remote sensing image, complex scene, attention mechanism, multi-scale feature fusion, ship detection

## Abstract

**Highlights:**

**What are the main findings?**

- An ES-YOLO framework combining EACSA attention, LSCD lightweight spatial–channel decoupling, and a hierarchical multi-scale design markedly improves multi-scale ship detection, especially for small and dense targets.
- The proposed model outperforms common detectors in challenging port scenes (occlusion, reflections, and clutter), delivering higher recall and overall mAP.

**What are the implications of the main findings?**

- Improved accuracy and robustness for ship detection in complex marine/port environments, supporting real-time maritime monitoring and port management.
- Establishes the TJShip benchmark and provides a practical methodology to guide future multi-scale ship detection research in complex port environments.

**Abstract:**

With the rapid development of remote sensing technology and deep learning, the port ship detection based on a single-stage algorithm has achieved remarkable results in optical imagery. However, most of the existing methods are designed and verified in specific scenes, such as fixed viewing angle, uniform background, or open sea, which makes it difficult to deal with the problem of ship detection in complex environments, such as cloud occlusion, wave fluctuation, complex buildings in the harbor, and multi-ship aggregation. To this end, ES-YOLO framework is proposed to solve the limitations of ship detection. A novel edge perception channel, Spatial Attention Mechanism (EACSA), is proposed to enhance the extraction of edge information and improve the ability to capture feature details. A lightweight spatial–channel decoupled down-sampling module (LSCD) is designed to replace the down-sampling structure of the original network and reduce the complexity of the down-sampling stage. A new hierarchical scale structure is designed to balance the detection effect of different scale differences. In this paper, a remote sensing ship dataset, TJShip, is constructed based on Gaofen-2 images, which covers multi-scale targets from small fishing boats to large cargo ships. The TJShip dataset was adopted as the data source, and the ES-YOLO model was employed to conduct ablation and comparison experiments. The results show that the introduction of EACSA attention mechanism, LSCD, and multi-scale structure improves the mAP of ship detection by 0.83%, 0.54%, and 1.06%, respectively, compared with the baseline model, also performing well in precision, recall and F1. Compared with Faster R-CNN, RetinaNet, YOLOv5, YOLOv7, and YOLOv8 methods, the results show that the ES-YOLO model improves the mAP by 46.87%, 8.14%, 1.85%, 1.75%, and 0.86%, respectively, under the same experimental conditions, which provides research ideas for ship detection.

## 1. Introduction

With the rapid development of remote sensing imaging technology and deep learning, ship detection based on remote sensing images has become an important research direction in the fields of marine monitoring [1,2,3,4,5,6], port management, maritime traffic supervision, disaster prevention and reduction, and military target recognition. As an important carrier of marine activities, the detection and identification of ships is of great significance for the reasonable development of marine resources, maritime security, and the maintenance of national maritime rights and interests. However, the complex imaging conditions, sea surface environment, and ship distribution characteristics make ship detection tasks face great challenges in remote sensing images. Ship targets vary significantly in size, shape, orientation, and illumination conditions, and are often disturbed by waves [7,8], sea fog, shadows, and background clutter, which significantly reduces the robustness of the model in detecting small ships, densely distributed ships, and low-contrast scenes.

With the continuous development of high-resolution remote sensing satellites and unmanned aerial vehicle (UAV) platforms, it is possible to obtain multi-source, multi-scale and high-precision image data, which provides rich data support for ship detection. The existing public datasets, such as HSRC2016 [9] and SeaShips [10], construct diverse samples in different seas and resolutions, which provide a basis for research. However, these datasets still have problems, such as concentrated ship scale distribution, insufficient attitude variation, and incomplete coverage of complex backgrounds, making it difficult to fully reflect the diversity and complexity of the real port environment. In addition, some studies have attempted to construct high-resolution ship detection datasets for specific scenes to make up for the shortcomings of existing samples in complex coastal environments [11]. However, there is still a lack of comprehensive data resources that take into account multi-scale, multi-type ships and multi-background scenes in general.

Ship detection methods have experienced the evolution from traditional artificial features to deep learning features. Early ship detection methods mainly rely on artificial features, such as HOG-SVM and Haar-like detectors [12,13], which are not robust in complex scenes. In recent years, the emergence of deep learning technology, especially Convolutional Neural Networks (CNN), has enabled the model to automatically extract multi-scale features from massive remote sensing images, which significantly improves the detection performance. The current mainstream deep learning detection methods are mainly divided into two categories: the two-stage method based on candidate boxes Faster R-CNN [14,15,16,17] and the end-to-end single-stage methods YOLO series [18,19,20,21,22], RetinaNet [23], etc. The former performs well in accuracy, but its inference speed is slow. The latter has significant advantages in detection efficiency and real-time performance, especially suitable for the rapid detection of large-scale remote sensing images. Despite the remarkable progress of deep learning methods, there are still several key problems in complex ocean scenes. Firstly, in terms of multi-scale feature fusion, most of the existing detection frameworks rely on a fixed feature pyramid structure [24,25] (Feature Pyramid Networks, FPN or Path Aggregation Network, PAN), and the feature transfer path is relatively single. It is difficult to fully integrate the semantic and detailed information of different scales, which leads to the weakening or even loss of small ship features after multiple down-sampling. Secondly, the robustness is still insufficient under complex background. The complex texture of the sea surface, severe illumination changes, and obvious wave reflection make the edges of the ship fuzzy and low contrast. When the texture of the ship and the background is similar, false detection and missed detection often occur. For example, in the harbor area, dock buildings, moored ships, and shadows are often misclassified as ships. Thirdly, the lack of context modeling ability limits the detection accuracy. The semantic recognition of ships not only depends on their own morphological features, but is also closely related to the scene elements such as surrounding waters, shoreline, and port facilities. If this environmental information is ignored, it is difficult for the model to correctly distinguish ships from non-ship structures in near-shore or complex backgrounds. Therefore, researchers have proposed a variety of contextual information enhancement strategies to improve the scene understanding ability of the model. Gong et al. [26] proposed Context-RoIs mechanism to dynamically generate extended regions around the target region to capture local spatial relationships. Wu et al. [27] designed a global context aggregation module (GCAM) to establish the semantic dependency between object and background in a wider range. The global boundary attention module (GB module) proposed by Wang [28] improves the detection accuracy in complex scenes by integrating multi-dimensional global features and local detail information. Chen et al. [29] proposed the CLGSA method to enhance the generalization ability of the model through cross-sample semantic correlation. These methods effectively enhance the detection performance in complex scenes but also bring the problems of computational overhead and structural complexity. In addition, Guan et al. [30] proposed an oriented SAR ship detection method based on edge deformable convolution and point set representation, which enhances the modeling capability of hull boundary structures through an edge-aware deformable sampling strategy and flexible geometric representation. This method effectively improves the detection robustness under significant ship rotation and morphological deformation. However, it is mainly designed for oriented target extraction in SAR scenarios and relies heavily on explicit geometric modeling. Its improvements do not directly address the multi-scale feature coupling, complex background suppression, and edge–detail semantic integration challenges in optical remote sensing ship detection tasks considered in this work. Therefore, how to achieve lightweight and real-time detection while maintaining high accuracy has become an important direction of current ship detection research. In recent years, attention mechanism and multi-scale feature fusion strategy have been widely introduced to enhance the feature adaptation ability and multi-scale expression ability of the model.

Aiming at the problems of insufficient multi-scale feature fusion, weak-edge information extraction ability, and significant complex background interference in ship detection in the above complex ocean environment, this paper conducts improvement research based on the YOLOv7 framework, aiming to improve the detection robustness and accuracy of the model in port and inshore scenes. The overall idea of the research is to enhance the comprehensive representation ability of the model for ship edge features, spatial details, and multi-scale semantic information while maintaining the lightweight and real-time performance of the network structure. To this end, this paper explores three aspects. Firstly, the edge and detail feature guidance mechanism is introduced to strengthen the response of the model to the boundaries and structural features of the ship, so as to reduce the interference of complex backgrounds such as waves and shadows. Secondly, an efficient decoupling strategy of spatial and channel features is designed to reduce feature redundancy and optimize the down-sampling process, so that the model can effectively transfer features between different scales. Thirdly, a multi-scale fusion hierarchical detection structure is constructed to balance the feature expression ability of large, medium, and small ship targets. Based on the self-built multi-scene remote sensing ship dataset TJShip, the proposed improvement idea is experimentally verified.

## 2. Materials and Methods

**Figure 1:**
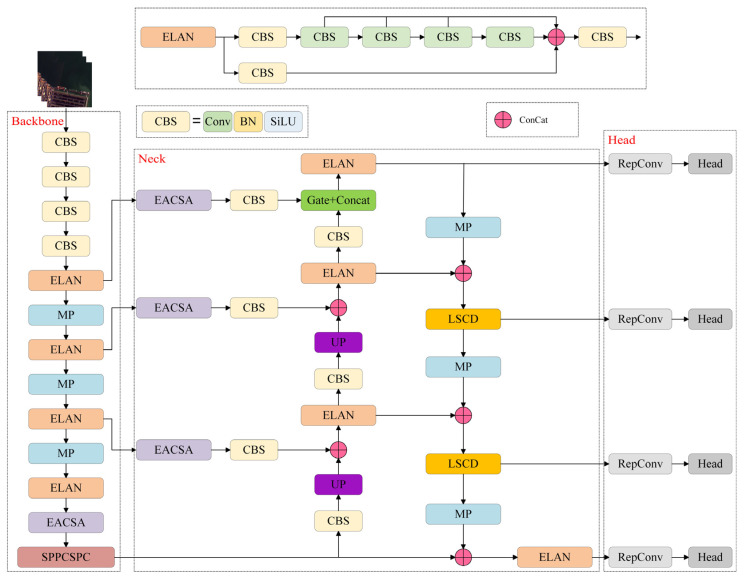
Overall framework of ES-YOLO. The network consists of three main components: (1) backbone, which extracts hierarchical semantic features from very low-level to high-level representations; (2) neck, where the proposed EACSA attention module enhances edge contours and suppresses background interference, while LSCD performs lightweight spatial–channel decoupled down-sampling; and (3) head, which generates final multi-scale detection outputs at four resolutions. The proposed modules work collaboratively to improve fine-grained ship boundary extraction, multi-scale feature fusion, and detection robustness in complex port scenes.

The overall architecture of the ES-YOLO model proposed in this paper is shown in Figure 1, which is composed of three parts: backbone, neck, and head. For the input image, the backbone network first processes the image and extracts hierarchical features, which represent very low, low, medium, and high-level semantic features, respectively. $X\in R^{H\times W\times 3}F_{i}\in R^{H_{i}\times W_{i}\times C_{i}},\;\left(i=1, 2,\;3, 4\right){\;F}_{1},F_{2},F_{3},F_{4}.$ The EACSA attention module of the neck network was used to extract the output features, and the edge information and detail feature information of the ship were extracted. In the down-sampling stage, the output information of the upper network is extracted through the LSCD module, and the calculation is effectively reduced by the separate convolution operation, while maintaining the extraction ability of key features, especially in the detection of complex backgrounds and small ships. Finally, the output (*i* = 1, 2, 3, 4) is obtained by the detection head. $D_{i}\in R^{\left(category,\;\;x,\;\;w,y,h\right)}$.

### 2.1. EACSA Module

**Figure 2:**
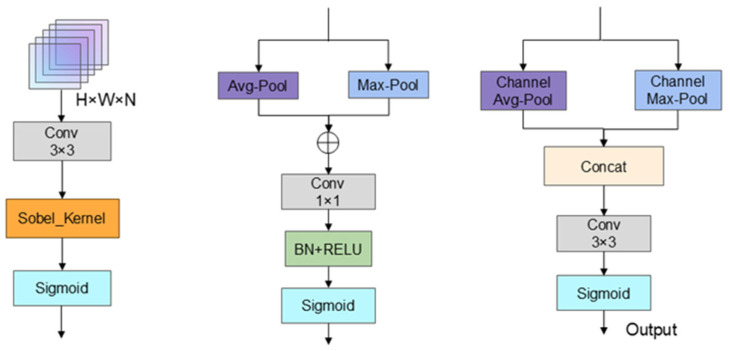
Structure of EACSA. EACSA integrates edge extraction, channel attention, and spatial attention to strengthen contour features and suppress background responses. The module enhances ship boundary perception without increasing large computational overhead.

In order to solve the problem that, with the traditional attention mechanism in ship detection tasks, in complex scenes, it is difficult to fully capture the structural information of the ship edge, and that it cannot effectively integrate channel and spatial dimensional features, this paper proposes an edge-aware multi-dimensional attention mechanism EACSA, as shown in Figure 2. The edge information is integrated into the channel and spatial attention module, and the edge structure information of the ship is extracted through the edge attention module to enhance the perception ability of the feature to the ship contour.

Given the input feature map as $X\in R^{H\times W\times 3}$, where H and W represent the height and width of the feature map, respectively, and the specified number of channels is 3, the edge detection convolutional layer uses depthwise separable convolution, and its convolution kernel is the predefined Sobel operator. This convolutional layer first performs a convolution operation on the input feature map to obtain the edge attention weight. Taking the absolute value of the feature map E gives $E_{abs}$, and then summing over the channel dimension yields a single-channel edge feature map $E_{sum}$. The edge attention weight $A_{age}$ is generated through the Sigmoid function. The edge attention weight is applied to the input feature map to obtain the edge-enhanced feature map $X_{age}$ (Equation (1)).

(1) $$X_{edge}=X\times \sigma (\sum_{c=1}^{C}E_{abs}^{c})$$

where $\sigma \left(\cdot \right)$ is the Sigmoid activation function, $E_{abs}^{c}$ denotes the *c*-th channel of $E_{abs}$, and $\in R^{H\times W\times 3}$ is the edge response map.

On the basis of edge enhancement, EACSA constructs a lightweight channel–spatial attention fusion structure. The channel attention module combines global average pooling and global Max pooling to capture the dependencies between channels. Secondly, a fully connected network composed of two 1 × 1 convolutional layers is used to generate channel attention weights. The channel attention weights are applied to the edge-enhanced feature maps to obtain the channel-enhanced feature maps $X_{channel}$ (Equations (2) and (3)).

(2) $$P=AdaptiveAvgPool2d\left(X_{edge}\right)+MaxPool2d\left(X_{edge},\left(H,W\right)\right)$$

(3) $$X_{channel}=X_{edge}\times \sigma (FC\left(P\right))$$

where $FC\left(\cdot \right)$ denotes the fully connected network.

The spatial attention module performs maximum pooling and average pooling in the channel dimension $X_{channel}$, and then generates spatial attention weights $A_{spatial}$ (Equation (4)) through a 3 × 3 convolutional layer and Sigmoid function after splicing. The spatial attention weights are applied to $\;X_{channel}$, and the enhanced feature map $X_{final}$ (Equation (5)) is obtained.

(4) $$A_{spatial}=\sigma \left(Conv3\times 3\left(Concat\left({max}_{c=1}^{C}X_{channel}^{c},\frac{1}{C}\sum_{c=1}^{C}X_{channel}^{c}\right)\right)\right)$$

(5) $$X_{final}=X_{channel}\times A_{spatial}$$

### 2.2. LSCD Module

**Figure 3:**
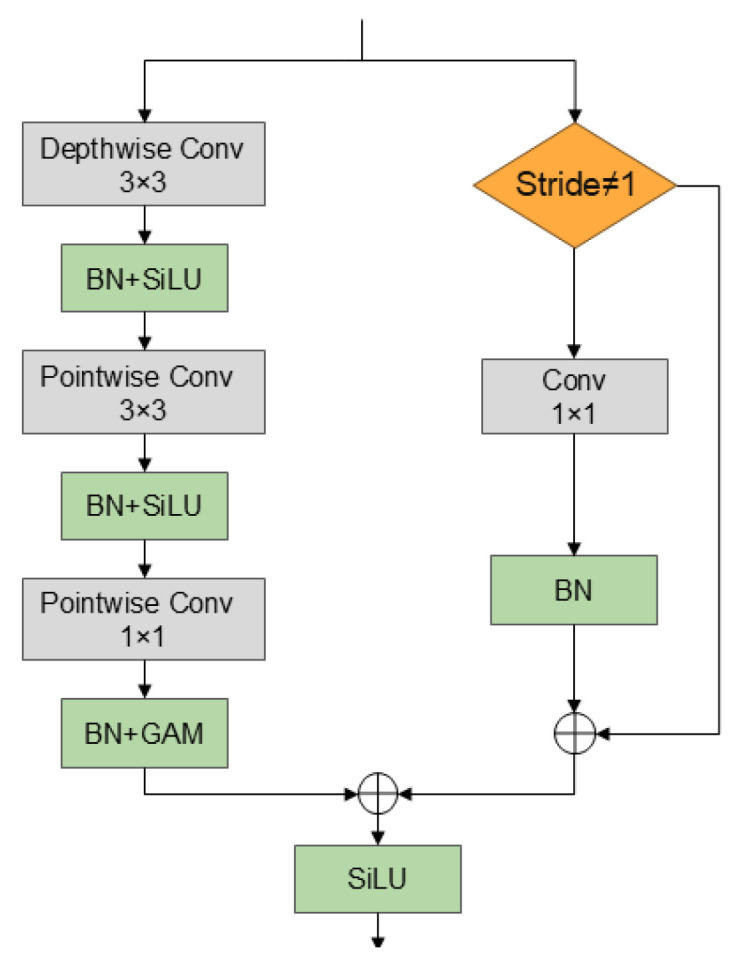
LSCD network structure. LSCD performs lightweight spatial–channel decoupled down-sampling to preserve discriminative features during resolution reduction. It improves small-ship feature retention while keeping computation efficient.

In the ship detection task, the efficient down-sampling module is extremely critical for the effective extraction and fusion of ship features at different scales. In this paper, a down-sampling module based on LSCD is proposed, as shown in Figure 3, with the help of spatial and channel decoupling design, lightweight attention mechanism GAM [31]. When dealing with ships of different scales, with the traditional down-sampling module, it is difficult to balance spatial detail preservation and channel semantic extraction. LSCD decouples spatial and channel operations to achieve refined feature processing. Let the input feature $X\in R^{B\times C\times H\times W}$. In the spatial decoupling operation, the depth separable convolution is used for spatial down-sampling, the convolution kernel size is $k_{s}$, and the step size is s. First, the depth separable convolution is performed, followed by batch normalization, and finally the activation function is used to obtain the feature $X_{s}$ (Equation (6)).

(6) $$X_{s}=SiLU\left(BN\left(DepthwiseConv2d\left({X,k}_{s},padding=\frac{k_{s}}{2},groups=C_{in}\right)\right)\right)$$

(7) $$X_{C1}=SiLU\left(BN\left(Conv2d\left(X_{s},C_{mid},1\right)\right)\right)$$

(8) $$X_{C2}=BN\left(Conv2d\left(X_{C1},C_{out},1\right)\right)$$

Through depthwise separable convolution, the computational complexity is reduced while the spatial details are preserved, and the excessive compression of spatial information by traditional convolution is avoided. In the channel decoupling operation, the spatially decoupled features are adjusted through two 1 × 1 convolutional layers for channel dimension adjustment, and the batch normalization and activation function are processed in the middle. Firstly, the number of channels was adjusted by the first 1 × 1 convolution, and then the batch normalization and activation function processing were performed to obtain $X_{C1}$ (Equation (7)), and then the second 1 × 1 convolution and batch normalization processing were performed to obtain $X_{C2}$ (Equation (8)). Among them, the feature interaction between channels is enhanced and the feature expression ability is improved by first reducing the dimension and then increasing the dimension $C_{mid}=max\left(\frac{C_{in}}{2},\frac{C_{out}}{2}\right)$.

In order to further highlight the characteristics of the ship and suppress the background noise, LSCD is embedded with a lightweight attention mechanism GAM. GAM calculates channel attention and spatial attention separately and multiplies them with input features. The channel attention is realized by global average pooling and 1 × 1 convolution and activation function, and the spatial attention is realized by 7 × 7 convolution, and finally $X_{attn}$ (Equation (9)) is obtained. The two methods combine to highlight the channel and spatial features of the ship.

(9) $$X_{attn}=X_{c2}\cdot ChannelAttention(X_{c2})\cdot SpatialAttention\left(X_{c2}\right)$$

(10) $$X_{res}=BN\left(Conv2d\left(X,C_{out},1,stride=s\right)\right)$$

In order to avoid information loss during down-sampling, LSCD introduces residual connection. When the number of input channels $C_{in}$ is inconsistent with the number of output channels $C_{out}$, the dimension of the input features X is adjusted by 1 × 1 convolution and batch normalization to obtain $X_{res}$ (Equation (10)). Then, the attention-processed features $X_{attn}$ and the adjusted residual features $X_{res}$ are added to obtain $X_{out}$, and finally the SiLU activation function is used to obtain $X_{out}$. The residual connection ensures the transmission of important features in the down-sampling process, alleviates the problem of gradient disappearance, and improves the stability of model training and feature extraction ability.

Unlike existing down-sampling operators (e.g., RepConv, DSConv, and GSConv), the proposed LSCD performs spatial and channel decoupling in two independent branches and then fuses them with a lightweight attention gate. This design differs from prior methods that only compress channel dimensions or only factorize the convolution kernel and is the core originality of our down-sampling modification.

### 2.3. Multi-Scale Structure

**Figure 4:**
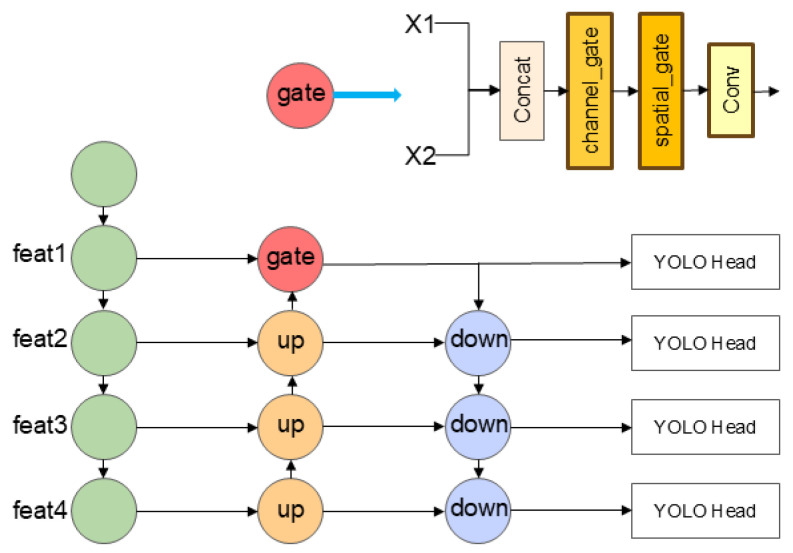
Multi-scale structure. Enhanced feature interaction across scales strengthens the detection of ships with large size variations. Channel–spatial gating further suppresses background noise during multi-scale information flow.

Aiming at the problem that YOLOv7 has insufficient detection ability for multi-scale ships, especially small ships in complex scenes, this paper introduces a hierarchical scale feature enhancement structure based on the original feature pyramid network PANet [32]. As shown in Figure 4, through cross-level feature fusion and a gating mechanism, a more refined multi-scale feature interaction path is constructed.

To avoid noise interference during cross-scale feature fusion, the Channel Spatial Gate module is introduced to dynamically adjust the contribution of features from different sources through the attention mechanism. At the scale fusion node, the global average/Max pooling of the channel dimension is performed on the input features to generate the channel attention weight. At the same time, spatial convolution is used to capture the local context and generate spatial attention masks. The gating mechanism outputs the weights between 0 and 1 through the Sigmoid function, adaptively selects the effective feature regions, and suppresses the interference of irrelevant background on the fusion features. For example, in small ship detection, the gating module preferentially activates the edge and contour information in the low-level features, which is complementary to the high-level semantic features to improve the ship positioning accuracy.

The input feature maps $X_{i}\in R^{B\times C\times H\times W}$ (*i* = 1, 2) are spliced through the gating mechanism, and the channel gating $G_{c}$ and spatial gating $G_{s}$ operations are performed in turn. Finally, the number of channels is adjusted through a 1 × 1 convolutional layer to make it the same as the total number of channels before splicing, and the final output is obtained as Y (Equations (11)–(13)).

(11) $$G_{c}=\sigma \left({Conv}_{1\times 1}^{C_{1}+C_{2}}\left(X^{\prime \prime }\right)\right)$$

(12) $$G_{s}=\left({Conv}_{7\times 7}\left(X_{C}\cdot G_{c}\right)\right)$$

(13) $$Y={Conv}_{1\times 1}^{C_{1}+C_{2}}\left(X_{C}\cdot G_{s}\right)$$

where $\;{X^{\prime }=Conv}_{1\times 1}^{\frac{C_{1}+C_{2}}{r}}\left(X\right)$, $X^{\prime \prime }=RELU\left(X^{\prime }\right)$ and $X_{C}$ is the feature map after splicing.

## 3. Data and Experimental Setup

### 3.1. Experimental Dataset Description

(1) TJShip dataset

Tianjin Port (north latitude 38°57′55″–39°10′50″, east longitude 117°31′50″–117°45′30″) is the largest comprehensive port in North China, with a total area of about 384 km^2^. Tianjin Port has a deep water channel, diversified berth system, and a complex ship activity scene, including cargo ships, passenger ships, fishing boats, engineering ships, and other ship types, which provide an ideal experimental area for carrying out ship monitoring research in the port.

**Figure 5:**
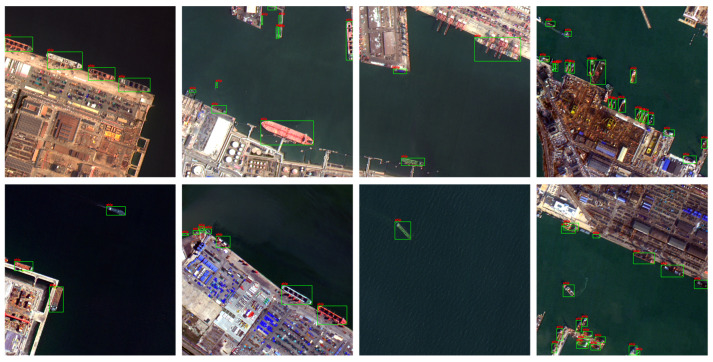
TJShip part of the training sample.

In this paper, the Gaofen-2 satellite is used as the data source to produce the TJShip dataset. Two GF-2 satellite remote sensing images on 5 December 2023 and 19 June 2024 with good weather conditions were selected as data sources. The acquired images were preprocessed, including radiometric calibration, atmospheric correction, orthographic correction, image cropping, and image fusion. The preprocessed images were cropped according to the size of 1024 × 1024, and the cropped data were augmented, including random flipping, random rotation, random scaling, etc., to increase the diversity of the training data. The TJShip dataset consisted of 1863 images, which were divided into a training set and test set according to 8:2. The specific information of the dataset is shown in Table 1 and Figure 5.

(2) SeaShips dataset

**Figure 6:**
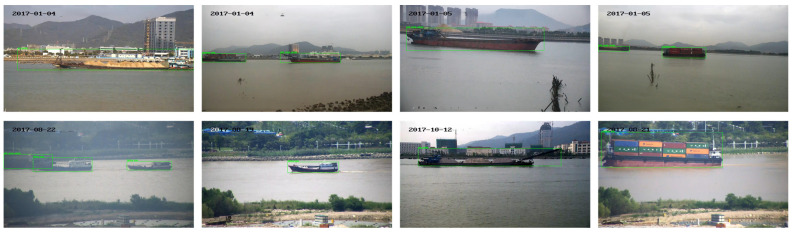
SeaShips part of the training sample.

The SeaShips [10] dataset is a representative large-scale public benchmark widely used in ship detection research, particularly suitable for port and near-shore surveillance scenarios. Developed by Dalian Maritime University, the dataset includes six typical ship categories—passenger ships, cargo vessels, fishing boats, engineering ships, oil tankers, and tugboats—captured from various real maritime monitoring environments. It features complex sea backgrounds, dense berth scenes, multi-scale targets, occlusions, and illumination variations, closely matching the characteristics of practical port surveillance imagery. All images are provided with accurate bounding-box annotations, enabling comprehensive evaluation of one-stage detection algorithms in challenging maritime conditions. As such, the SeaShips dataset serves as a fundamental benchmark for validating ship detection performance in port scenarios. The specific information of the dataset is shown in Figure 6.

### 3.2. Experimental Design

The experiment was run on Ubuntu 18.04, the server was equipped with Intel (R) Xeon (R) Gold 6430 processor, and the graphics card was NVIDIA GeForce RTX 4090 GPU. The training and testing environment is constructed based on CUDA 11.3 and PyTorch 1.10.0 framework. In the training process, the training parameter set includes the number of iterations (epoch), batch size (batch size), initial learning rate (lr0), final learning rate (lr1), momentum, etc. The epoch was set to 100, the batch size was set to 4, the lr0 was set to 1 × 10^−3^, the lr1 was set to 1 × 10^−5^, and the momentum was set to 0.937 [33]. The IoU threshold was set to 0.5, and the SGD optimizer was used to update the network model parameters iteratively. The input image size is uniformly scaled to 1024 × 1024.

(1) Ablation experiments

In order to evaluate the enhancement effects of EACSA module, LSCD module, and multi-scale structure on YOLO model, eight ablation experiments (none of which used pre-trained model) were designed. The detailed configurations are shown in Table 2.

In order to quantitatively evaluate the impact of reduction in EACSA module on detection performance (precision, recall, mAP, and F1), and find the optimal compression ratio setting between accuracy and efficiency, four groups of experiments are designed, as shown in Table 3.

In order to quantitatively compare the influence of five activation functions of LeakyReLU, GeLU, PReLU, SiLU, and ReLU on the detection performance (precision, recall, mAP, and F1) in the LSCD down-sampling module, five groups of experiments were designed, and the detailed experimental parameters are shown in Table 4.

(2) Comparative experiments

In order to evaluate the performance of ES-YOLO in ship detection, Faster R-CNN, RetinaNet, YOLOv5, and YOLOv8 are selected as the comparison models, and the parameters are shown in Table 5.

### 3.3. Evaluation Metrics

In this paper, precision, recall, mAP and F1 scores are used to evaluate the accuracy of ship recognition. Recall (Equation (14)) measures the ability of the model to correctly identify all actual positive examples in the recognition task, which is the ratio of the number of positive examples identified by the model to the number of positive examples that actually exist.

(14) Recall=TP/(TP+FN)

where TP is the score predicted by the model as a ship which is also the score actually as a ship; FP is the fraction of the model that incorrectly identifies other classes as ships; and FN is the part of the model that incorrectly identifies other objects as ships.

Accuracy (Equation (15)) reflects the reliability of the model’s prediction for positive samples, which is the proportion of samples that the model identifies as positive samples that are actually positive.

(15) Precision=TP/(TP+FP)

The F1 score (Equation (16)) intuitively reflects the trade-off effect of the model in accurately identifying positive samples and avoiding false positives and is a kind of weighted average of precision and recall.

(16) $$F1=2\times \left(Precision\times Recall\right)/(Precision+Recall)$$

mAP (Equation (17)) is the average of the average detection accuracy of all classes, where the threshold for IoU is set to 0.5.

(17) $$mAP=\sum_{i=1}^{n}AP_{i}/n$$

## 4. Experimental Results and Analysis

### 4.1. Ablation Experiments

**Figure 7:**
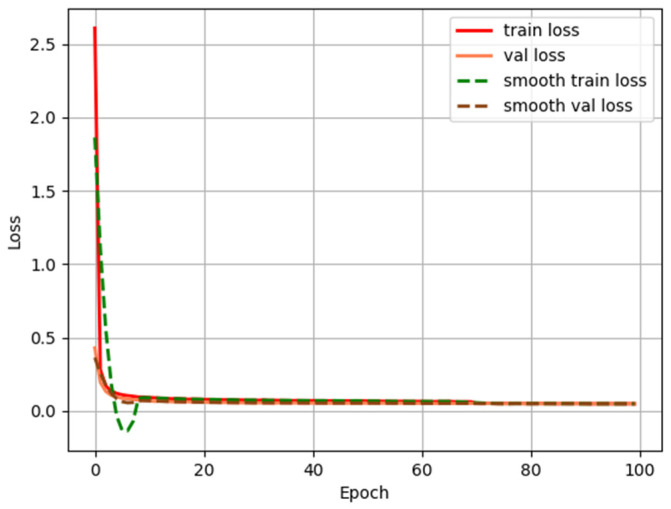
Training set and validation set loss values during model training.

The accuracy evaluation results of ablation experiments are shown in Table 6, and the loss changes during the training of the ES-YOLO model are shown in Figure 7.

Figure 7 shows the changes in the training set and validation set loss of the ES-YOLO model within 100 epochs. It can be seen that the training set loss and validation set loss values are decreasing, and the validation set loss value starts to level off at a certain state, indicating that the model is trained normally.

(1) EACSA

In the EACSA module, the channel attention realizes the adaptive recalibration of channel features through a two-layer convolution structure, and the compression ratio is used to control the dimension reduction ratio of the channel to achieve a balance between model complexity and feature expression ability. The experimental results are shown in Table 7.

The experimental results show that the compression ratio has a significant impact on the model checking performance. When reduction is 4, more feature information is retained, but the redundancy between channels is high, and the attention distribution is not concentrated. When reduction is 16 or 32, the channel information is excessively compressed, and the key semantic features are lost, resulting in a decrease in detection accuracy. Comprehensive comparison shows that when reduction is 8, the model achieves 91.54% in precision, 70.77% in recall, 82.74% in mAP, and 80% in F1, respectively, and the overall performance is the best. Moderate channel compression can not only reduce the computational burden but also enhance the ability of the attention module to focus on key features, making the model more stable in small target detection and complex background scenes. Therefore, reduction to 8 can be used as the best balance setting between detection accuracy and model complexity for EACSA module.

In order to verify the influence of EACSA module on the performance of the model, this module is introduced on the base model for comparative experiments, and the results are shown in Table 6. After the introduction of EACSA, the overall detection performance of the model is improved, in which precision is increased to 94.01%, recall is increased to 72.38%, mAP is increased to 84%, and F1 value is increased to 82%. From the results, the precision is increased by 2.9%, indicating that the false detection rate of the model is significantly reduced in complex backgrounds. mAP was increased by 0.83%, indicating that the overall detection accuracy was optimized. At the same time, recall is increased by 1.6%, which reflects the enhanced detection ability of the model in weak-target or low-contrast scenes. This shows that the EACSA module plays a positive role in feature extraction and salient region focusing. The reason for the performance improvement is that the EACSA module enhances the responsiveness of the model to key features by introducing a joint attention mechanism in the channel and spatial dimensions. Channel attention uses the fusion of global average pooling and maximum pooling to adaptively allocate channel weights, which effectively strengthens semantic salient features and suppresses irrelevant background information, thereby reducing false detections and improving precision. Spatial attention captures salient regions in the two-dimensional space, so that the model can more accurately locate the edge and contour structure of the ship in the complex sea background, thereby improving recall and F1 value. In addition, Sobel convolution enhancement, an edge-aware mechanism embedded in EACSA, further strengthens the gradient response of the target boundary and improves the adaptability of the model to blurred contours, uneven illumination, and wave interference scenes. This synergistic effect of edge enhancement and attention focusing makes the model have stronger environmental robustness while maintaining high detection accuracy.

(2) LSCD

To investigate the impact of nonlinear activations on the proposed LSCD architecture, five commonly used activation functions (LeakyReLU, GeLU, PReLU, SiLU, and ReLU) were evaluated under identical network configurations and training settings. The results are summarized in Table 8. Among them, ReLU achieves the highest detection accuracy, with precision, recall, mAP, and F1 reaching 92.53%, 70.46%, 83.56%, and 0.80, respectively. PReLU and GeLU obtain comparable performance but remain slightly inferior to ReLU in all metrics, while SiLU and LeakyReLU show a noticeable decline in both recall and mAP.

Although SiLU is the default activation in YOLOv7, its smooth nonlinear compression weakens the response at object boundaries, resulting in insufficient preservation of fine-grained features for small ships. In contrast, the hard-threshold property of ReLU introduces stronger activation sparsity, effectively suppressing sea surface noise and enhancing the feature contrast between ships and background regions. This behavior is particularly beneficial in the down-sampling stage, where edge-aware decoupling is employed. Moreover, the residual and multi-branch fusion structure of LSCD mitigates the neuron inactivation issue commonly associated with ReLU, ensuring stable gradient flow during optimization.

Overall, the results indicate that activation selection is highly architecture dependent. In our LSCD framework, ReLU provides a more favorable trade-off between gradient sparsity, edge preservation, and convergence stability, leading to superior detection performance in complex maritime scenes.

In order to evaluate the influence of LSCD module on the performance of the model, LSCD is introduced into the basic model for comparison test. The experimental results are shown in Table 6. When only LSCD module is introduced, the precision, recall, mAP, and F1 values of the model are increased to 93.74%, 71.46%, 83.71%, and 81%, respectively. The LSCD module reduces the computational cost of feature down-sampling while maintaining accuracy and improves the detection efficiency of the model while maintaining the accuracy of the model. The LSCD module introduces a feature mapping method that decouples space and channel, so that the down-sampling process can focus on spatial structure and semantic information, respectively, avoiding the information loss problem caused by feature coupling in traditional convolution down-sampling. At the same time, the lightweight design inside the LSCD module can improve the accuracy of feature selection while reducing the amount of parameters and calculations, which makes the model perform better in small ship detection and complex background suppression.

In addition, the stable contribution of LSCD can be further verified from the results of the combination with other modules. When LSCD and EACSA are used at the same time, the mAP of the model is improved by about 0.63% compared with the Baseline, indicating that the two have complementary advantages in the feature extraction and down-sampling stage. When LSCD is combined with the multi-scale structure, the model improves the recall significantly, indicating that the LSCD module can effectively enhance the retention ability of features at different scales and further improve the recall performance of detection.

In summary, the LSCD module significantly improves the information expression quality of the feature down-sampling stage under the premise of keeping the overall model size unchanged and improves the feature retention and complex background suppression effect of small ships. It is an important part of the ES-YOLO framework to achieve efficient feature extraction and performance improvement.

(3) Multi-scale

In order to verify the influence of the multi-scale structure on the performance of the model, this paper introduces the multi-scale structure on the basis of the benchmark model for comparison tests. The experimental results are shown in Table 6. The precision, recall, mAP, and F1 values of the model reach 91.03%, 72.62%, 84.23%, and 81%, respectively. Among them, the precision is slightly decreased compared with the baseline model, while the recall and mAP are significantly improved, especially the mAP, which is increased by about 1.1%, indicating that the multi-scale structure can significantly enhance the detection ability of the model on objects of different scales. It can be seen from the results that the multi-scale structure effectively improves the detection performance of small objects. The core idea is to enable the model to simultaneously obtain high-level semantic information and low-level detailed features through the parallel multi-scale feature pathway, so as to improve the diversity and complementarity of the feature space. Compared with the traditional single-path feature transfer method, the problems of the decline of spatial resolution of high-level features and the insufficient semantic expression of low-level features after multiple down-sampling are effectively alleviated. The improvement of recall indicates that the model is more sensitive in capturing fine-grained targets such as small ships, and the overall improvement of mAP reflects that multi-scale information fusion enhances the robustness of the model in complex scenes. The precision slightly decreases, which is caused by the redundancy of some features or the introduction of noise in the scale fusion process, but the overall F1 value is still higher than that of the baseline model, indicating that the multi-scale structure effectively improves the comprehensiveness and robustness of detection while maintaining the overall detection accuracy.

**Figure 8:**
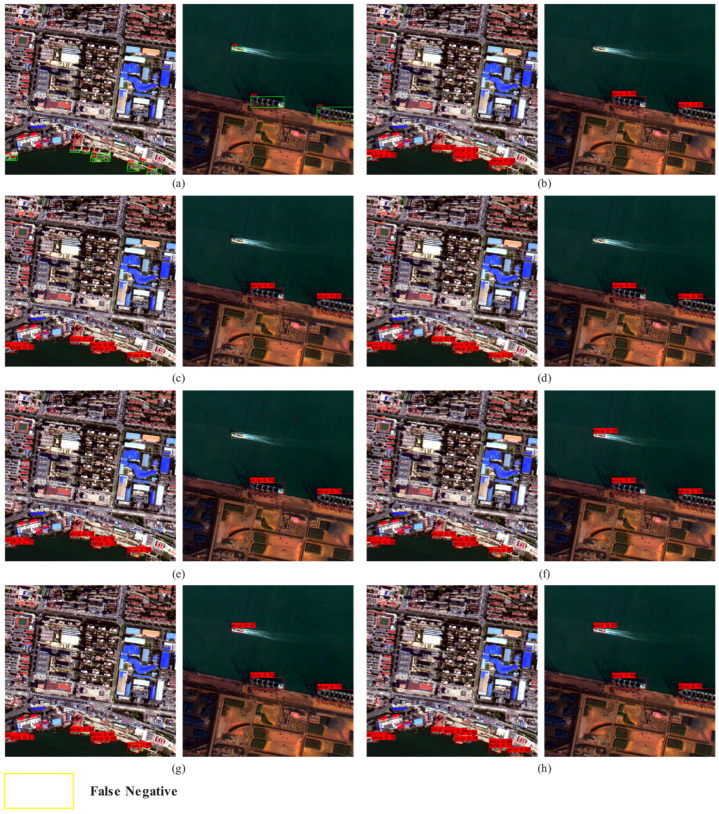
Results of ablation experiments. (**a**) Label; (**b**) +EACSA; (**c**) +LSCD; (**d**) +Multi; (**e**) +EACSA + LSCD; (**f**) +EACSA + Multi; (**g**) +LSCD + Multi; (**h**) ES-YOLO.

**Figure 9:**
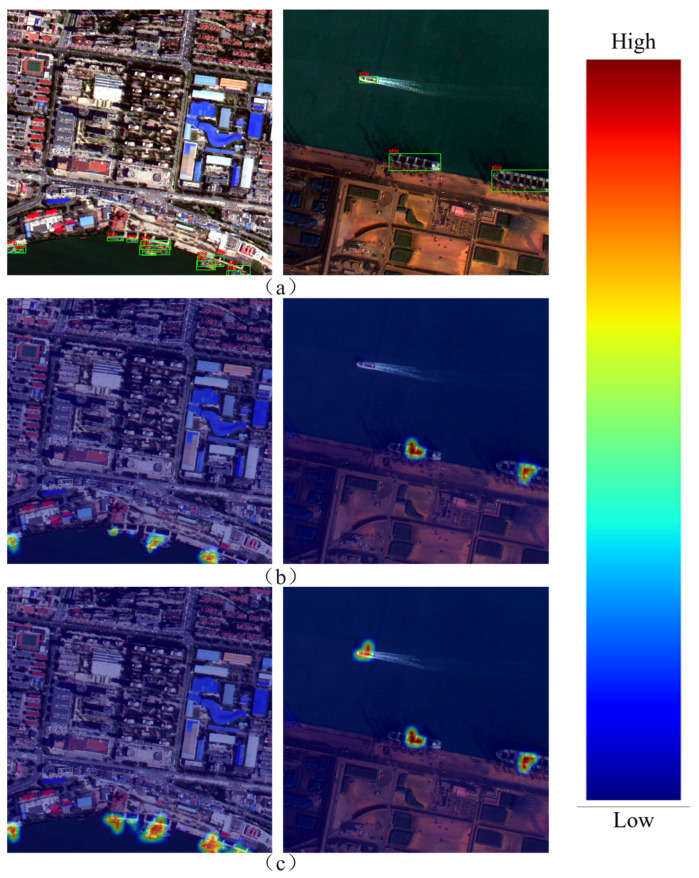
Heat map visualization. (**a**) Original image; (**b**) feature map of the original image; (**c**) the feature map after ES-YOLO processing.

In addition, in order to more intuitively illustrate the improvement of model performance by each module, this paper conducts visual analysis. As shown in Figure 8, where (a) represents the real labeling results, (b)–(h) correspond to the detection results after removing or adding different modules, respectively, the red box represents the detection results, and the yellow box represents the missed detection. It can be seen from the Figure that when the key module is missing, the model is prone to problems such as incomplete detection boxes and missed detection in complex backgrounds or dense ship areas. With the gradual introduction of the improved module, the detection results are gradually closer to the real annotation, which can better cover the ship targets in different scales and complex environments and significantly reduce the missed detection phenomenon. Figure 9 presents the heatmap visualization results of ES-YOLO and the baseline model. High-activation regions correspond to ship bodies and boundary structures, indicating strong feature responses, whereas low-activation regions mainly appear in sea clutter and non-target areas. This visualization confirms that the designed modules not only enhance the representation of ship-related features but also effectively suppress background responses, thereby improving detection robustness in complex maritime scenes.

### 4.2. Comparative Experiments

In order to prove the effectiveness of ES-YOLO method, it is compared with three other object detection algorithms: Faster R-CNN, RetinaNet, YOLOv5, and YOLOv8. The detection performance on TJShip dataset is shown in Table 9.

As shown in Table 9, the proposed model algorithm shows significant advantages in all performance metrics. The precision, recall, mAP, and F1 of ES-YOLO reach 94.16%, 73.89%, 84.92%, and 82%, respectively, and the ship recognition accuracy is the highest. The recognition accuracy of Faster R-CNN network is the lowest, which is due to the fact that Faster R-CNN is a two-stage detection method with a complex structure and slow inference speed, which is not conducive to efficient detection under complex background and multi-scale ship. Compared with YOLOv5 and YOLOv8, with relatively high accuracy, ES-YOLO has an accuracy increased by 1.42% and the recall rate increased by 7.04%. Due to the limited capability of feature fusion and context modeling, the detection performance of small ships and dense ships in complex port scenes is still insufficient. ES-YOLO greatly improves its feature extraction ability through the introduction of EACSA and significantly improves the recall ability of small ships through multi-scale structure. Although RetinaNet has a certain ability to detect ships with high accuracy, the recall is only 65.92%, indicating that most real ships have not been detected.

**Figure 10:**
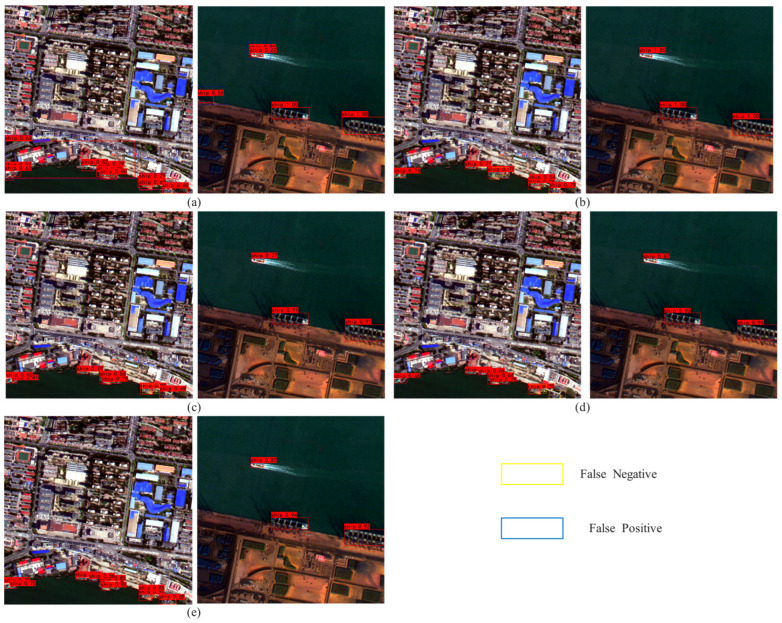
Compares the experimental results. (**a**) Faster R-CNN; (**b**) RetinaNet; (**c**) YOLOv5; (**d**) YOLOv8; (**e**) ES-YOLO.

In order to visually show and compare the detection performance of various algorithms, this paper carries out a visual analysis, and the results are shown in Figure 10. Figure 10a–e correspond to the visualization of the detection results of Faster R-CNN, RetinaNet, YOLOv5, YOLOv8, and ES-YOLO, respectively. Figure 10a shows that Faster R-CNN has a large number of false detections and missed detections, which further illustrates that the complex two-stage algorithm is not suitable for ship detection in complex scenes. It can be seen from Figure 10b that RetinaNet has achieved good detection accuracy on some ships with no background occlusion and relatively complete hull, but there are still many missed detections. YOLOv5 and YOLOv8 achieve a certain balance between detection accuracy and recall rate, while ES-YOLO, proposed in this paper, can still accurately identify multi-scale ship targets under complex backgrounds, improve the detection effect of small ships, improve the detection accuracy, and obtain excellent detection performance.

**Figure 11:**
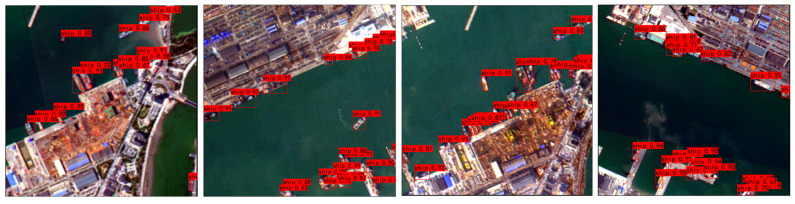
Results under dense conditions.

In addition to the quantitative comparison, we further verify the robustness of ES-YOLO under challenging maritime scenarios. Although no dedicated quantitative subset is provided for extreme-density or weather-specific evaluation, we show in Figure 11 that the proposed method remains robust under dense berth scenes, wake interference, and low-contrast illumination conditions.

These visual examples provide complementary evidence that ES-YOLO maintains consistent detection behavior in real-world degraded environments.

### 4.3. Generalization Experiments

**Figure 12:**
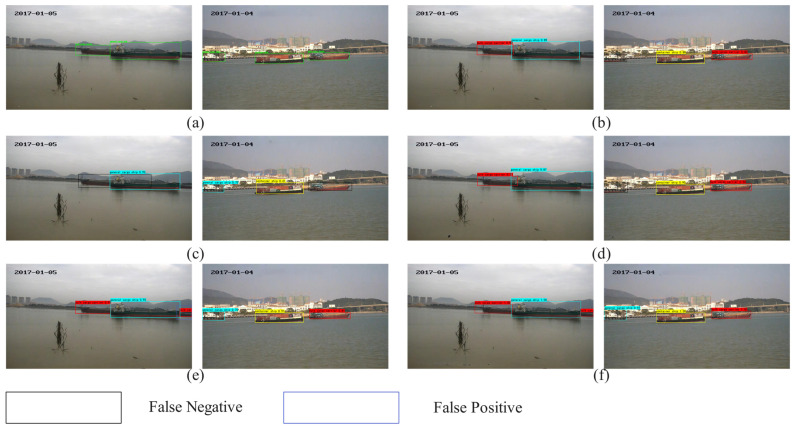
Generalized experimental results. (**a**) Label; (**b**) Faster R-CNN; (**c**) RetinaNet; (**d**) YOLOv5; (**e**) YOLOv8; (**f**) ES-YOLO.

According to the quantitative results in Table 10 and the mAP comparison shown in Figure 12, clear performance differences can be observed among various detectors in port-scene ship detection. Overall, one-stage detectors achieve high accuracy while maintaining real-time inference capabilities. YOLOv8 and ES-YOLO exhibit particularly strong performance, achieving near-saturated accuracy across almost all categories, with mAP values of 97.50% and 97.83%, respectively. Notably, ES-YOLO achieves nearly perfect accuracy for bulk cargo and ore carriers, indicating that its enhanced feature representation is highly effective at capturing large ship structures while suppressing ocean-surface background interference.

In comparison, Faster R-CNN demonstrates stable performance, but its accuracy decreases for small-scale categories such as fishing boats and passenger ships. This suggests that the traditional two-stage framework still has limitations in localizing fine-grained targets under complex port backgrounds. YOLOv5 shows balanced performance overall, but its accuracy remains lower than the more advanced YOLOv8 family.

A prominent observation is the significant performance drop of RetinaNet in the ore carrier category, where the accuracy falls to only 0.22, resulting in a relatively low overall mAP of 74.66%. This indicates that RetinaNet’s feature pyramid struggles with large-scale variations and elongated ship structures commonly found in port environments.

In summary, the experimental results demonstrate that advanced one-stage detectors (ES-YOLO) significantly outperform both traditional two-stage models and earlier one-stage approaches in port-scene ship detection. Their superior robustness to multi-scale targets, densely berthed ship clusters, and visually complex maritime backgrounds make them more suitable for practical deployment in real-world port monitoring systems.

## 5. Discussion

In order to solve the problems of ship detection in optical remote sensing images, such as the difficulty of small target detection, strong complex background interference, and significant scale change, an improved single-stage detection model named ES-YOLO is proposed. The model uses YOLOv7 as the basic framework and effectively improves the detection accuracy and robustness in complex ocean and port scenes through structure optimization and feature enhancement mechanism. Specifically, the edge-aware Channel-Spatial Attention Module (EACSA) is introduced to pay attention to both the edge details and semantic feature channels of the target in the feature extraction process, which significantly enhances the feature expression ability of the model for small ship targets. A lightweight spatial–channel decoupled down-sampling module (LSCD) is designed to reduce the computational complexity and realize the efficient fusion of multi-level features, which improves the detection efficiency and information transmission ability of the model. In addition, a hierarchical multi-scale structure is used to fully exploit the correlation and complementarity between features at different scales, so as to enhance the adaptability of the model to scale changes and complex backgrounds.

Experimental results on the TJShip dataset show that the ES-YOLO model proposed in this paper is superior to the mainstream detection algorithms in multiple evaluation indicators. Compared with the original YOLOv7 model, the ES-YOLO model has the mAP improved by about 1.75%, the precision improved by about 3.06%, and the recall improved by about 3.89%. Compared with the classical detection networks such as YOLOv5, RetinaNet, and Faster R-CNN, the mAP of ES-YOLO is increased by 46.87%, 8.14%, and 1.85%, respectively, which verifies that ES-YOLO has better detection performance and stronger generalization ability in complex port, dense occlusion and strong reflection scenes. The practicability and robustness of the proposed method under complex background conditions are proven.

The ES-YOLO model proposed in this paper significantly improves the accuracy and stability of ship target detection while maintaining a high detection speed by introducing feature enhancement and multi-scale fusion mechanism, which provides an efficient and reliable technical approach for small target detection in high-resolution remote sensing images. However, there is still room for further improvement in the detection ability of the model in extremely dense distribution, severe occlusion, and multi-class mixed scenes. Robustness under weather degradation and severe occlusion is an important future direction. A dedicated benchmark containing stratified weather conditions and density-level annotations will be integrated in future work to further validate ES-YOLO. Robustness under weather degradation and severe occlusion is an important future direction. A dedicated benchmark containing stratified weather conditions and density-level annotations will be integrated in future work to further validate ES-YOLO.

## Figures and Tables

**Table 1:** Details of TJShip dataset.

Metrics	Numerical Values
Total image count	1863 images (1024 by 1024)
Total number of targets	7110 (4,863,112 pixels)~
Average number of targets per image	3.82 (min 1, Max 53)
Small target (area ≤ 1024)	2987
Medium target (1024 < area ≤ 9216)	3101
Large Target (area > 9216)	1022

**Table 2:** Ablation experiment parameter Table.

Network	Description	Dataset	Epoch	Batch Size	Optimizer
YOLOv7	Basic network	TJShip	100	4	SGD
EACSA-YOLO	YOLO + EACSA	TJShip	100	4	SGD
LSCD-YOLO	YOLO + LSCD	TJShip	100	4	SGD
Multi-YOLO	YOLO + Multi	TJShip	100	4	SGD
E-S-YOLO	YOLO + EACSA + LSCD	TJShip	100	4	SGD
E-M-YOLO	YOLO + EACSA + Multi	TJShip	100	4	SGD
S-M-YOLO	YOLO + LSCD + Multi	TJShip	100	4	SGD
ES-YOLO	YOLO + EACSA + LSCD + MUlti	TJShip	100	4	SGD

**Table 3:** Experimental parameter table of compression ratio.

Reduction	Dataset	Epoch	Batch Size	Optimizer
4	TJShip	100	4	SGD
8	TJShip	100	4	SGD
16	TJShip	100	4	SGD
32	TJShip	100	4	SGD

**Table 4:** Compression ratio experimental parameter table.

Method	Description	Dataset	Epoch	Batch Size	Optimizer
LeakyReLU	The negative interval gradient is retained on the basis of ReLU to avoid neuron deactivation.	TJShip	100	4	SGD
GeLU	Smooth activation based on Gaussian distribution to improve nonlinear expression ability.	TJShip	100	4	SGD
PReLU	The negative half-axis slope can be learned to enhance feature adaptability.	TJShip	100	4	SGD
SiLU	Silu smooths nonlinear functions with both gradient stability and feature continuity.	TJShip	100	4	SGD
ReLU	Piecewise linear activation, sparse and efficient, and fast convergence.	TJShip	100	4	SGD

**Table 5:** Comparative experimental parameter table.

Network	Dataset	Epoch	Batch Size	Optimizer	Backbone
Faster R-CNN	TJShip	100	4	SGD	ResNet-50
RetinaNet	TJShip	100	4	SGD	MobileNet
YOLOv5	TJShip	100	4	SGD	CSPDarknet53
YOLOv8	TJShip	100	4	SGD	C2f
ES-YOLO	TJShip	100	4	SGD	CSPDarknet53

**Table 6:** Results of ablation experiments.

	EACSA	LSCD	Multi-Scale	P	R	mAP	F1
Baseline	×	×	×	0.9110	0.7000	0.8317	0.80
	√	×	×	0.9401	0.7238	0.8400	0.82
	×	√	×	0.9374	0.7146	0.8371	0.81
	×	×	√	0.9103	0.7262	0.8423	0.81
	√	√	×	0.9229	0.7000	0.8380	0.80
	√	×	√	0.9252	0.7331	0.8486	0.82
	×	√	√	0.9104	0.7115	0.8396	0.80
	√	√	√	0.9416	0.7389	0.8492	0.82

Blue indicates the second largest value of this column, while red represents the maximum value. P, R, and mAP are rounded to four decimal places, while F1 is rounded to two decimal places. √ means to use the module, and × means not to use the module.

**Table 7:** Effect of different compression ratios on EACSA.

Method	P	R	mAP	F1
R = 4	0.9136	0.6992	0.8253	0.79
R = 8	0.9154	0.7077	0.8274	0.80
R = 16	0.9144	0.7069	0.8269	0.80
R = 32	0.9115	0.6969	0.8282	0.79

**Table 8:** Influence of different activation functions on the down-sampling structure.

Method	P	R	mAP	F1
LeakyReLU	0.9123	0.6885	0.8246	0.78
GeLU	0.9211	0.6915	0.8236	0.79
PReLU	0.9222	0.6931	0.8295	0.79
SiLU	0.9134	0.6900	0.8232	0.79
ReLU	0.9253	0.7046	0.8356	0.80

Blue indicates the second largest value of this column, while red represents the maximum value; P, R, and mAP keep four decimal places, and F1 keeps two decimal places.

**Table 9:** Performance comparison of different methods.

Method	P	R	mAP	F1
Faster R-CNN	0.3488	0.4685	0.3805	0.40
RetinaNet	0.9376	0.6592	0.7678	0.77
YOLOv5	0.9274	0.7077	0.8307	0.80
YOLOv8	0.9415	0.6685	0.8406	0.78
ES-YOLO	0.9416	0.7389	0.8492	0.82

Blue indicates the second largest value of this column, while red represents the maximum value. P, R, and mAP are rounded to four decimal places, while F1 is rounded to two decimal places.

**Table 10:** Performance comparison of different methods on SeaShips.

AP	Bulk Cargo Carrier	Container Ship	Fishing Boat	General Cargo Ship	Ore Carrier	Passenger Ship	mAP
Faster R-CNN	0.97	0.98	0.92	0.97	0.97	0.92	0.9541
RetinaNet	0.64	0.95	0.85	0.90	0.22	0.90	0.7466
YOLOv5	0.91	0.97	0.93	0.91	0.90	0.92	0.9229
YOLOv8	0.97	0.98	0.98	0.98	0.98	0.96	0.9750
ES-YOLO	0.99	0.98	0.97	0.98	0.99	0.96	0.9783

Blue indicates the second largest value of this column, while red represents the maximum value. mAP are rounded to four decimal places, while AP is rounded to two decimal places.

## Data Availability

For the private TJShip dataset, requests for access can be directed to caolixiang1@stumail.nciae.edu.cn.
